# Biofilm Formation in Clinical *Acinetobacter baumannii* Is Influenced by Isolate Source and Is Inversely Correlated With Antibiotic Resistance

**DOI:** 10.1155/bmri/9348199

**Published:** 2026-01-16

**Authors:** Qutaiba Ababneh, Dua’a Alawneh, Ziad Jaradat, Esra’a Al-Zoubi, Ali Atoom, Neda’a Aldaken, Ekhlas Al-Rousan, Yazeed Alshari, Ismail Saadoun

**Affiliations:** ^1^ Department of Biotechnology and Genetic Engineering, Faculty of Science and Arts, Jordan University of Science and Technology, Irbid, Jordan, just.edu.jo; ^2^ Department of Medical Laboratory Sciences, Al-Ahliyya Amman University, Amman, Jordan, ammanu.edu.jo; ^3^ Department of Applied Biology, College of Science, University of Sharjah, Sharjah, UAE, sharjah.ac.ae

**Keywords:** *Acinetobacter baumannii*, antibiotic resistance, biofilm, extensively drug resistant, intensive care unit

## Abstract

*Acinetobacter baumannii* is a troublesome bacterium that is highly prevalent in hospital settings, particularly in intensive care units (ICUs). Biofilm is one of the main virulence factors that makes *A. baumannii* a successful pathogen, enabling it to survive the harshest environments. This study aimed to corrolate the biofilm‐forming capacity of clinical *A. baumannii* isolates with their antibiotic resistance phenotypes and isolation sources. A total of 327 clinical isolates originated from different hospitals, were recovered from diverse clinical specimens collected from patients admitted to the ICU and non‐ICU wards. The isolates were characterized for their resistance phenotypes and biofilm formation capacities. Most *A. baumannii* isolates showed high resistance patterns against all examined antibiotics. Based on the resistance profiles, 81.2% and 12.3% of isolates were classified into extensively resistant (XDR) and multidrug‐resistant (MDR), respectively. Moreover, the number of the ICU isolates exhibiting the XDR phenotype (86.7%) was higher than non‐ICU XDR isolates (76.4%). The biofilm‐forming capacity varied among the isolates, with most of the isolates forming either strong (44.3%) or weak biofilms (25.7%). Additionally, the fraction of ICU isolates with a strong capacity to form biofilms (60.7%, 91/150) was higher compared with the non‐ICU isolates (30.5%, 54/177). We found a significantly higher tendency to form biofilms in isolates that are susceptible to 10 out of the 17 antibiotics (*p* = 0.014–0.002), including three carbapenems. In addition, a significant difference in the ability to form biofilms was revealed between the isolates originating from different hospitals and clinical specimens. Notably, a higher tendency to form biofilms was associated with susceptible strains isolated from blood (*p* = 0.024–0.04) and cerebrospinal fluid (*p* = 0.001–0.009). Our findings indicate that investigating the biofilm formation capacity of clinical *A. baumannii* strains could help identify patients requiring short or extended therapeutic treatments.

## 1. Introduction

*Acinetobacter baumannii* has emerged as a persistent and multidrug‐resistant pathogen responsible for community‐ and hospital‐acquired infections worldwide. This bacterium is responsible for opportunistic ventilator‐associated pneumonia, urinary tract infections, blood and intra‐abdominal infections, meningitis, endocarditis, surgical site infections of the skin and soft tissues, and osteomyelitis, ophthalmitis, and keratitis [1]. Due to its formidable capacity to resist antimicrobial drugs and host defenses, the World Health Organization announced *A. baumannii* as one of the most serious ESKAPE organisms (*Enterococcus faecium*, *Staphylococcus aureus*, *Klebsiella pneumoniae*, *A. baumannii*, *Pseudomonas aeruginosa*, and *Enterobacter* species) that exhibit a high tendency to resist the effects of antibiotics [2].

Most importantly, *A. baumannii* acquires new genetic resistance determinants at a high rate via mutational changes, as well as by horizontal gene transfer of plasmids, transposons, and integrons from other members of the species, genus, and/or the Enterobacteriaceae family [3]. This acquisition of new resistance determinants is amplified by the selective pressure of the hospital environment, as well as the crowding of healthcare settings, the lack of hygiene, and the increase in worldwide travel [4, 5]. Healthcare physicians are currently left with few effective therapeutic options to combat *A. baumannii*, and despite the extensive efforts aimed at discovering novel antibiotics effective against these pathogens, only a few novel antibiotics have been reported, and therapeutic options remain scarce [6].

In addition to antibiotic resistance, biofilm formation is an important feature that renders *A. baumannii* a successful pathogen and complicates treatment of *A. baumannii* infections [7, 8]. The formation of biofilms coincides with changes in gene expression and metabolism within the biofilm microbial community. Such changes may confer resistance to antibiotics and host immune system mechanisms of clearance [9]. Furthermore, biofilm structures provide an optimal platform for the exchange of genetic material between the different microbial cells within the biofilm community [10]. Consequently, this may further restrict therapeutic options.

The capacity of *A. baumannii* to form biofilms on diverse abiotic surfaces present in healthcare settings, as well as medical devices and equipment, plays a major role in causing nosocomial infections, especially pneumonia and catheter‐associated urinary tract infections [8, 11]. In addition, *A. baumannii* has been shown to have the ability to adhere to and colonize cell surfaces, such as erythrocytes [12] and human alveolar and bronchial epithelial cells [13], thus enhancing its ability to cause infections of the lung and blood.

The factors contributing to biofilm‐forming capacity are strain‐dependent and are not yet fully determined [14, 15]. Therefore, understanding the role of these factors might improve infection control procedures in healthcare settings. Previous studies attempted to find a correlation between the ability of clinical isolates to form biofilms and their antibiotic resistance [16, 17]. However, the findings of these studies are controversial and were limited by the number of isolates and/or the factors analyzed. In this study, we investigated the relationship between antibiotic resistance phenotypes and biofilm‐forming ability of 327 clinical *A. baumannii* isolates originating from three hospitals, recovered from different infection sites, and isolated from intensive care unit (ICU) and non‐ICU patients.

## 2. Materials and Methods

### 2.1. Bacterial Isolates

A total of 327 nonreplicate, clinical *A. baumannii* isolates were recovered from diverse clinical specimens from patients admitted to three major local hospitals in Jordan: Al‐Bashir Hospitals (BH), King Abdullah University Hospital (KAUH), and New Zarqa Governmental Hospital (ZH). The majority of isolates were recovered from the following clinical specimens: urine, sputum, wound swabs, blood, cerebrospinal fluid, and abdominal swabs. All isolates were identified as *A. baumannii* biochemically using the VITEK 2 automated system. Ethical approvals for this study were obtained from the Institutional Review Board (IRB) of Jordan University of Science and Technology (JUST) under Approval Number 14/111/2017. Furthermore, permission to collect the isolates was also obtained from the IRB of Jordan Ministry of Health (JMOH) under Approval Number 180030, and the collection was performed according to the MOH regulations.

### 2.2. Molecular Identification of the Isolates

Molecular confirmation of the identity of the collected isolates was performed by PCR amplification of the internal regions of *bla_OXA-51_* [18] and a multiplex PCR assay described previously. Genomic DNA was extracted using the Genomic DNA Purification kit (ZymoResearch, United States) following the manufacturer′s recommendation. All amplification reactions consisted of 1x the ready‐to‐use mix (Intron, Korea), 0.4 pmol/*μ*L of each primer and 20 ng/*μ*L of template DNA. The PCR conditions were as follows: initial denaturation at 94°C for 5 min, followed by 25 cycles of denaturation at 94°C for 30 s, annealing at 65°C for 30 s, extension at 94°C for 45 s, and a final extension step at 72°C for 7 min. *A. baumannii* ATCC 19606 and *Escherichia coli* ATCC BAA‐25922 were included as positive and negative controls, respectively.

### 2.3. Antibiotic Susceptibility Testing

Antibiotic resistance profiles for all isolates were determined using the Kirby–Bauer disc diffusion method [19] for 16 different antibiotics representing eight families following Clinical and Laboratory Standards Institute guidelines (CLSI, 2021). All the following antibiotic discs were purchased from Oxoid (United Kingdom): tetracycline (TE, 30 *μ*g), sulfamethoxazole–trimethoprim (SXT, 5 *μ*g), piperacillin/tazobactam (TZP, 10 *μ*g), ampicillin/sulbactam (10 *μ*g), doripenem (10 *μ*g), imipenem (10 *μ*g), meropenem (10 *μ*g), ciprofloxacin (5 *μ*g), levofloxacin (5 *μ*g), ceftriaxone (30 *μ*g), ceftazidime (30 *μ*g), cefepime (30 *μ*g), ampicillin (10 *μ*g), amikacin (30 *μ*g), tobramycin (10 *μ*g), and gentamicin (10 *μ*g). An inoculum of 1.5 × 10^8^ CFU/mL was swabbed onto Mueller–Hinton agar plates (Oxoid, United Kingdom), the discs were added, and plates were incubated at 37°C for 16–18 h. The following day, the diameters of the inhibition zones were measured, and the isolates were classified as MDR, XDR, or PDR based on the criteria followed by Magiorakos et al. [20].

### 2.4. Biofilm Assay

A microtiter plate method was adopted to determine the biofilm‐forming capacity of *A. baumannii* isolates, as described previously [21]. Bacterial cells were incubated in LB broth (Oxoid, United Kingdom) at 37°C for 18 h. Cultures were inoculated into a 96‐well flat‐bottomed polystyrene microtiter plate (three wells for each isolate). The plates were incubated aerobically without shaking at 37°C for 24 h. Sterile LB broth was included in all plates as a negative control, while *A. baumannii* ATCC19606 was used as a positive control [22]. After the incubation period, the planktonic growth in the wells was gently removed, the plates were washed three times with phosphate‐buffered saline (pH 7.2), and then air dried. The dried adherent bacterial cells in the wells were stained with 0.1% *w*/*v* of crystal violet for 20 min. The stain was removed gently, followed by washing the plates four times with distilled water and the microtiter plates were dried at room temperature. The dried crystal violet was solubilized by the addition of 95% ethanol, and the optical density was measured at 570 nm using an ELISA reader (BioTek Epoch). The biofilm assay for each isolate was conducted in three wells with the whole experiment was repeated three times. The average OD values were calculated and compared with the cut‐off OD (ODc) to determine the biofilm phenotype as follows: nonbiofilm producer: OD ≤ ODc; weak biofilm producer: ODc < OD ≤ 2 × ODc; moderate biofilm producer: 2 × ODc < OD ≤ 4 × ODc; or strong biofilm producer: OD > 4 × ODc [23].

### 2.5. Statistical Analysis

Chi‐square or Fisher′s exact tests with odds ratio were used (Version 15, SAS, North Carolina) to determine the statistical significance of the data. A *p* value of < 0.05 was considered significant.

## 3. Results

### 3.1. Characteristics and Molecular Identification of the Isolates

A total of 150 isolates were recovered from clinical specimens collected from patients admitted to the ICUs, while 177 isolates were recovered from patients admitted to the general wards. The demographics of the isolates investigated in this study are shown in Table 1. Half of the isolates were collected from BH/Amman (50.6%), 35.8% from KAUH/Irbid, and 13.6% from the ZH/Zarqa. The isolates were mostly recovered from the sputum (44.3%) and urine (10.4%), followed by blood (9.8%), wound (8%), cerebrospinal fluid (5.2%), pus (4.6%), and abdominal swabs (3.4%). The remaining 14.4% of isolates were recovered from minor sources such as foot, hip‐joint fluid, endotracheal tube, testis, and thigh swabs (Table 1).

**Table 1:** Demographics of the *A. baumannii* isolates investigated in this study.

	**ICU**	**Non-ICU**	**Total**
Male to female			
Hospital	150	177	327
KAUH	41	75	116
ZH	21	26	47
BH	88	76	164
Clinical specimen type			
Abdominal swab	2	9	11
Blood	16	16	32
Cerebrospinal fluid	6	11	17
Sputum	91	54	145
Urine	7	27	34
Wounds	2	24	26
Pus	5	10	15
Other	15	32	47

Abbreviations: BH, Al‐Bashir Hospitals; KAUH, King Abdullah University Hospital; ZH, New Zarqa Governmental Hospital.

### 3.2. Antibiotic Susceptibility Testing

**Figure 1:**
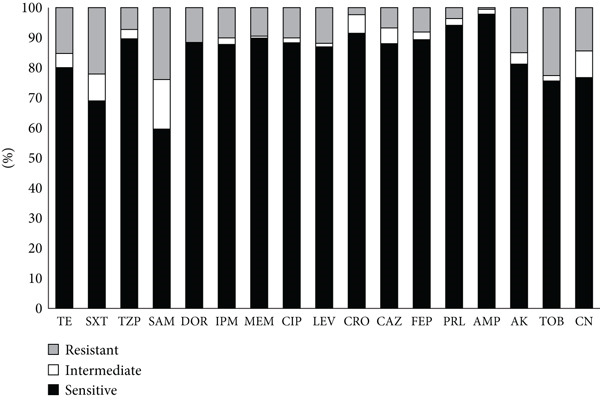
Antibiotic susceptibility of *A. baumannii* isolates. Stacked bars show the proportion of isolates classified as resistant (R), intermediate (I), and susceptible (S) to each agent by disc diffusion testing, interpreted according to CLSI guidelines. The *y*‐axis indicates percentage of isolates. TE, tetracycline; SXT, trimethoprim–sulfamethoxazole; TZP, piperacillin–tazobactam; SAM, ampicillin–sulbactam; DOR, doripenem; IPM, imipenem; MEM, meropenem; CIP, ciprofloxacin; LEV, levofloxacin; CRO, ceftriaxone; CAZ, ceftazidime; FEP, cefepime; PRL, piperacillin; AMP, ampicillin; AK, amikacin; TOB, tobramycin; CN, gentamicin.

Antibiotic susceptibility patterns for all isolates were determined against 17 different agents representing eight classes of antibiotics using the disc diffusion method (Figure 1). Most *A. baumannii* isolates showed high resistance patterns. As a general trend, the ICU isolates were more resistant than the non‐ICU isolates for all agents. The average percentage of the resistant ICU isolates among the 17 antibiotics was 89.1%, compared to only 80% for resistant non‐ICU isolates. Ampicillin and piperacillin were the least effective agents, with 97.9% and 92.7% of the isolates resistant to these antibiotics, respectively. On average, resistance to carbapenems (meropenem, doripenem, and imipenem) was observed in 88.7% of the isolates, with the ICU isolates (94.4%) exhibiting a higher resistance than the non‐ICU isolates (83.8%). Overall, 89.6% of the isolates were resistant to TZP, 83.4% to ciprofloxacin, 89.3% to cefepime, 86.9% to levofloxacin, 88.1% to ceftazidime, 81% to amikacin, 79.8% to TE, 76.8% to gentamicin, and 75.5% to tobramycin. The combinational drugs, SXT, and ampicillin–sulbactam were the most effective among all other antibiotics used in this study, as only 68.8% and 59.3% of isolates exhibited the resistance phenotype against these two agents, respectively. Resistance to these two agents was higher in the ICU isolates, as 76.7% and 61.3% of the isolates exhibited resistance to SXT and ampicillin–sulbactam, respectively, compared to 62.1% and 57.6% of the non‐ICU isolates.

Based on the obtained antibiotic susceptibility patterns and the classification system established by [20], 81.2% of the isolates were classified as extensively drug resistant (XDR), whereas 12.3% were MDR (Figure 2a). As indicated by Figure 2b,c, the number of the ICU isolates exhibiting the XDR phenotype (86.7%) was higher than non‐ICU, XDR isolates (76.4%).

Figure 2Resistance–phenotype distribution in *A. baumannii* isolates. Charts show the proportion of isolates classified as non‐MDR, MDR, and XDR. (a) All isolates. (b) ICU isolates (*n* = 150). (c) Non‐ICU isolates (*n* = 177). MDR, multidrug resistant; XDR, extensively drug resistant.

**Figure:**
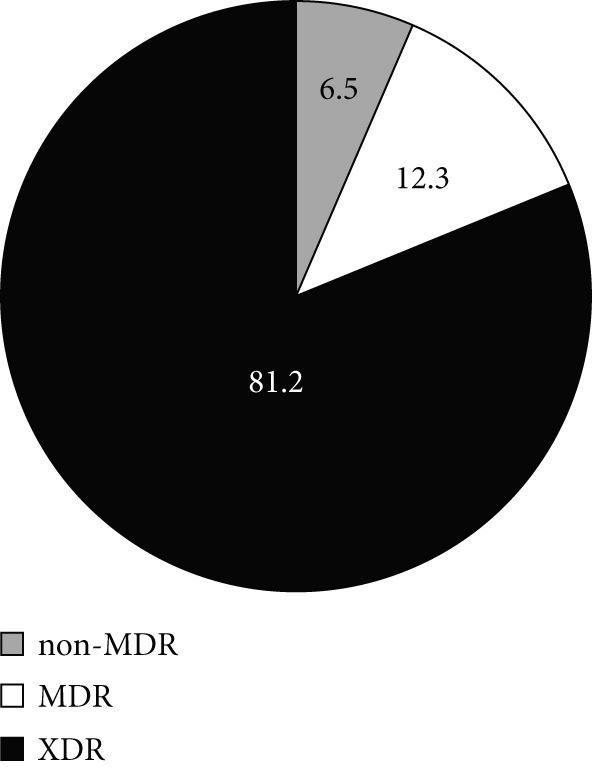
(a)

**Figure:**
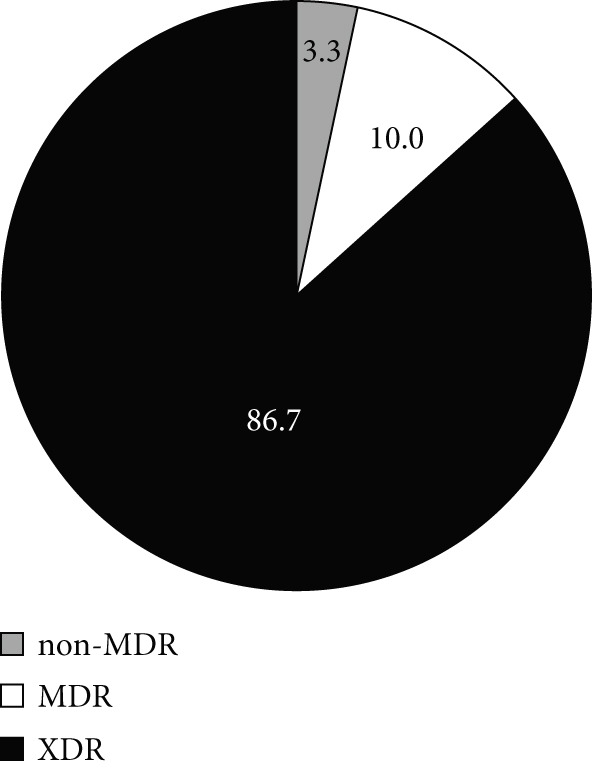
(b)

**Figure:**
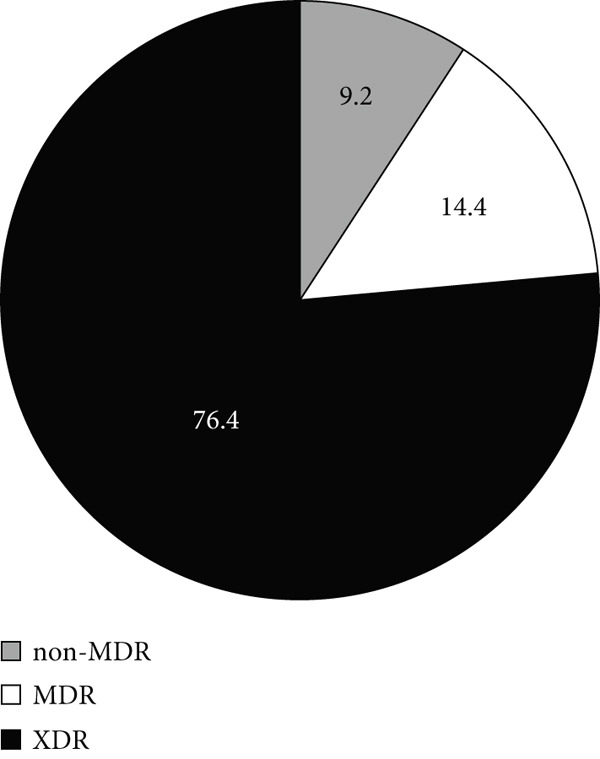
(c)

### 3.3. Biofilm Formation Ability

The distribution of isolates according to their biofilm formation phenotypes is shown in Figure 3. Most isolates (82.3%) were biofilm formers, while only 17.7% were nonbiofilm formers. The biofilm‐forming capacity varied among the isolates, as most isolates formed either strong or weak biofilms and only 12.2% (*n* = 40) formed moderate biofilms (Figure 3a). As indicated by Figure 3b,c, the fraction of ICU isolates with a strong capacity to form biofilms (60.7%, 91/150) was higher than that of the non‐ICU isolates (30.5%, 54/177).

Figure 3Biofilm formation in clinical *A. baumannii* isolates. Charts show the proportion of isolates categorized as strong, moderate, weak, or non‐biofilm formers by crystal‐violet microtiter assay. (a) All isolates. (b) ICU isolates (*n* = 150). (c) Non‐ICU isolates (*n* = 177).

**Figure:**
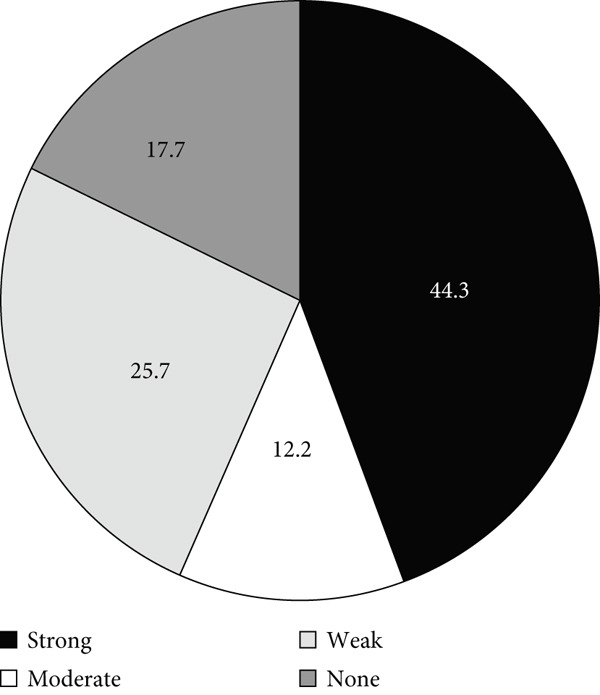
(a)

**Figure:**
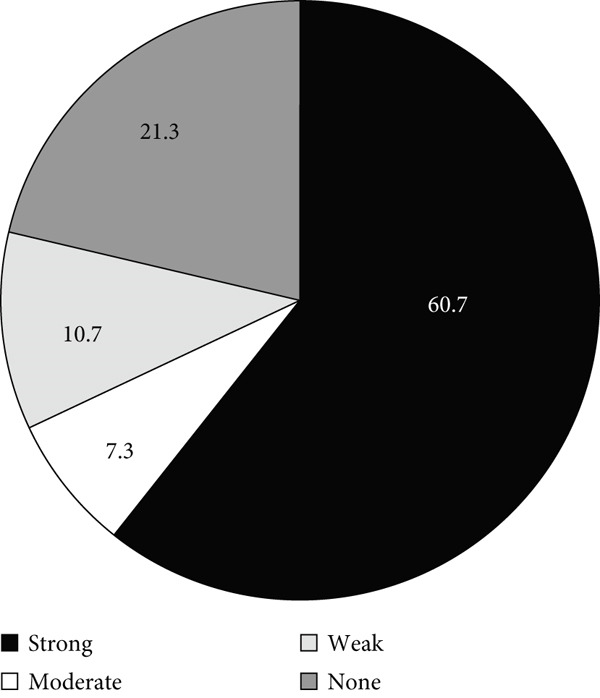
(b)

**Figure:**
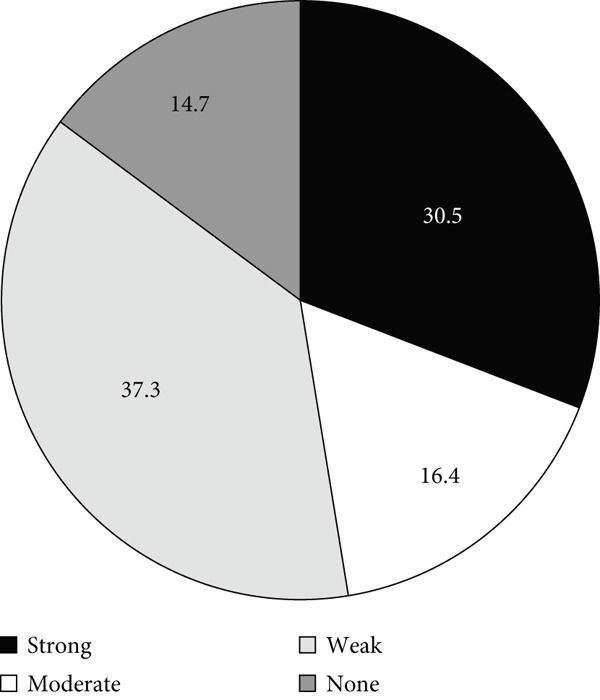
(c)

### 3.4. Correlation Between Biofilm Formation and the Source of the Isolates

**Figure 5:**
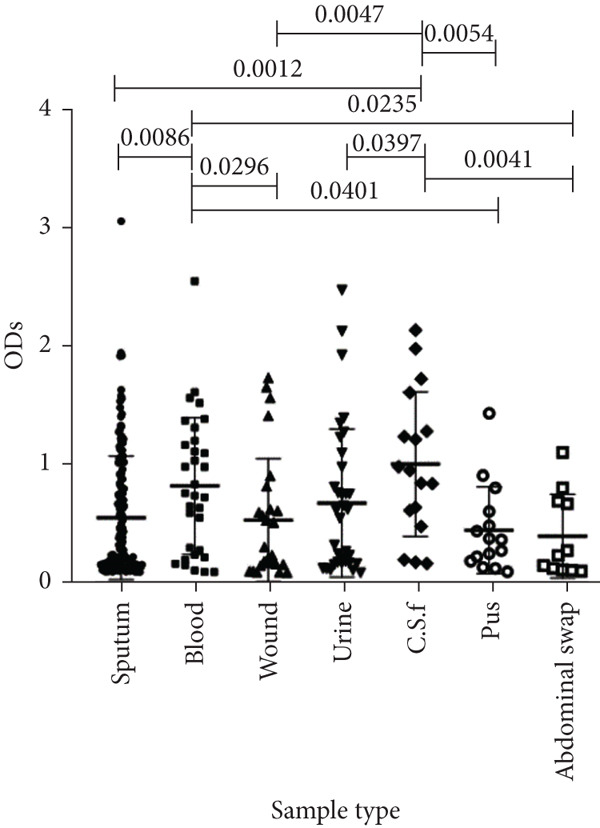
Biofilm burden by clinical specimen type. Crystal‐violet microtiter assay OD_570_ values for isolates from sputum, blood, wound, urine, CSF, pus, and abdominal swab. Each dot is one isolate, and horizontal lines indicate mean ± SD (same *y*‐axis across panels).

To determine whether the biofilm formation capacity is correlated more with the ICU or non‐ICU isolates, the ODs obtained from the biofilm assay for both types of isolates were compared using Fisher′s exact test (Figure 4). No significant difference was observed in the biofilm formation capacity between the ICU and non‐ICU isolates (*p* = 0.145), as well as the male and female isolates (*p* = 0.786). Using the same type of statistical analysis, we found that the isolates from the AlBahir and AlZarqa hospitals tended to form stronger biofilms than the isolates collected from KAUH. However, the capacity to form biofilms in isolates from AlBahsir and AlZarqa hospitals was similar. With respect to the type of clinical specimens from which the *A. baumannii* isolates were recovered, the highest tendency to form biofilm was in the CSF and blood isolates (Figure 5). The isolates recovered from CSF samples exhibited a significantly higher capacity to form biofilms than the isolates from all other sample types except for the blood isolates (*p* = 0.001–0.009). Similarly, a significantly higher biofilm‐forming capacity was observed in the isolates recovered from blood samples compared to sputum, wound, pus, and abdominal swab isolates (*p* = 0.024–0.04).

Figure 4Biofilm burden by patient type, sex, and hospital. Crystal‐violet microtiter assay OD_570_ values are shown as dot plots. Each dot is one isolate, and horizontal lines indicate mean ± SD (same *y*‐axis across panels). (a) Patient type (ICU vs. non‐ICU). (b) Gender (male vs. female). (c) Hospitals (BH, KAUH, and ZH). BH, Al‐Bashir Hospitals; KAUH, King Abdullah University Hospital; ZH, New Zarqa Governmental Hospital.

**Figure:**
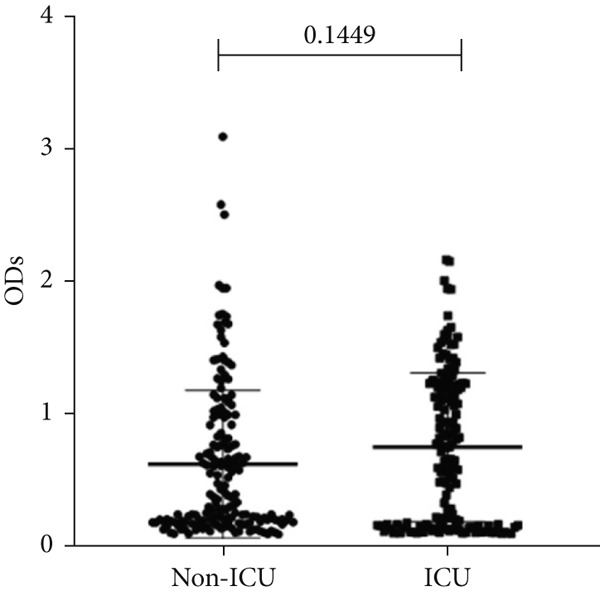
(a)

**Figure:**
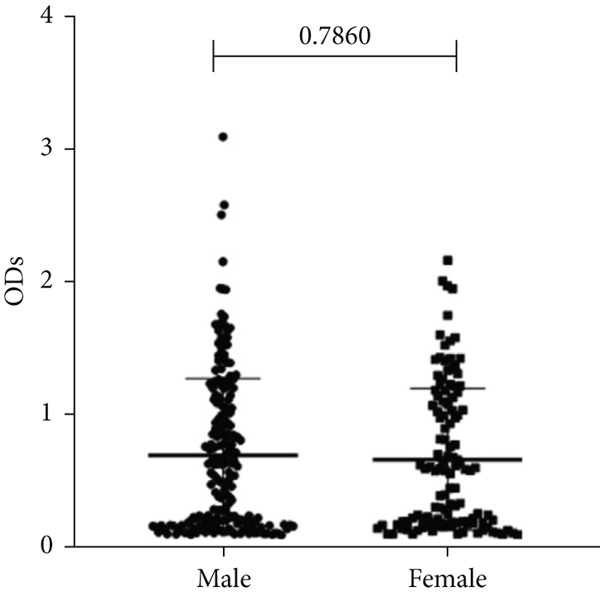
(b)

**Figure:**
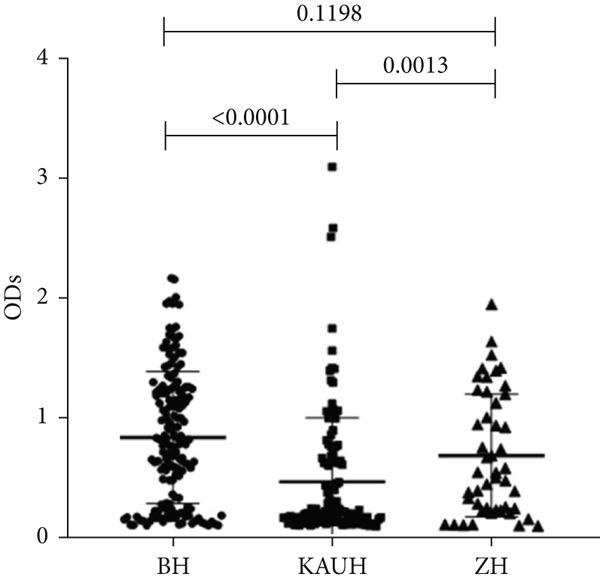
(c)

### 3.5. Correlation Between Biofilm Formation and Antibiotic Resistance

**Figure 7:**
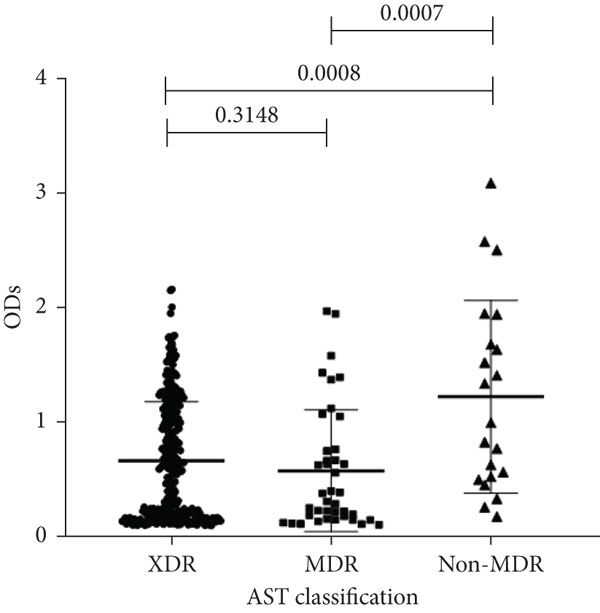
Biofilm burden by resistance phenotype. Crystal‐violet microtiter OD_570_ values for isolates classified as XDR, MDR, and non‐MDR. Each dot is one isolate, and horizontal lines indicate mean ± SD (same *y*‐axis across panels). MDR, multidrug resistant; XDR, extensively drug resistant.

To determine whether biofilm formation is correlated with resistance to certain types of antibiotics, we compared the biofilm former capacity of all isolates with respect to 17 agents. Following the susceptibility testing for each agent, the isolates were grouped into susceptible and nonsusceptible, with the latter group including the isolates with the resistant and intermediate phenotypes. As shown in Figure 6, a higher tendency to form biofilms was observed in the isolates that were susceptible to the following 10 antibiotics: ampicillin–sulbactam, doripenem, imipenem, meropenem, SXT, TZP, levofloxacin, cefepime, ceftazidime, and ciprofloxacin (*p* = 0.014–0.002). For the remaining agents, no significant difference in the ability to form biofilms was found between the susceptible and nonsusceptible isolates. When we tested the correlation of resistance with the ICU and non‐ICU isolates alone, a significantly higher tendency to form biofilms was observed in the susceptible ICU isolates for all antibiotic agents except for ceftriaxone (data not shown). Finally, non‐MDR isolates exhibited a higher tendency to form biofilms than the MDR (*p* < 0.001) and XDR (*p* < 0.001) isolates (Figure 7).

Figure 6Biofilm burden by susceptibility to individual antibiotics. (a–q) For each agent, crystal‐violet microtiter OD_570_ values are shown for isolates classified as nonsusceptible (intermediate or resistant) versus susceptible by CLSI guidelines. Each dot is one isolate, and horizontal lines indicate mean ± SD (same *y*‐axis across panels). (a) Amikacin, (b) gentamicin, (c) tobramycin, (d) ampicillin–sulbactam, (e) piperacillin–tazobactam, (f) doripenem, (g) imipenem, (h) meropenem, (i) cefepime, (j) ceftazidime, (k) ceftriaxone, (l) ciprofloxacin, (m) levofloxacin, (n) trimethoprim–sulfamethoxazole, (o) ampicillin, (p) piperacillin, and (q) tetracycline.

**Figure:**
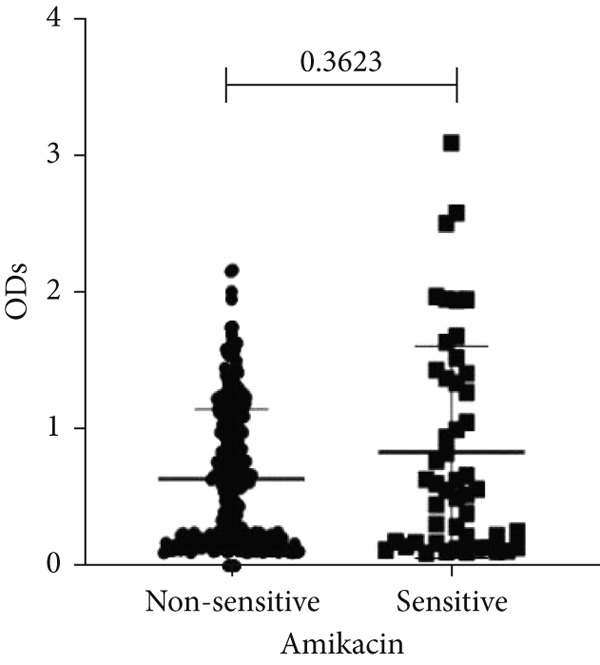
(a)

**Figure:**
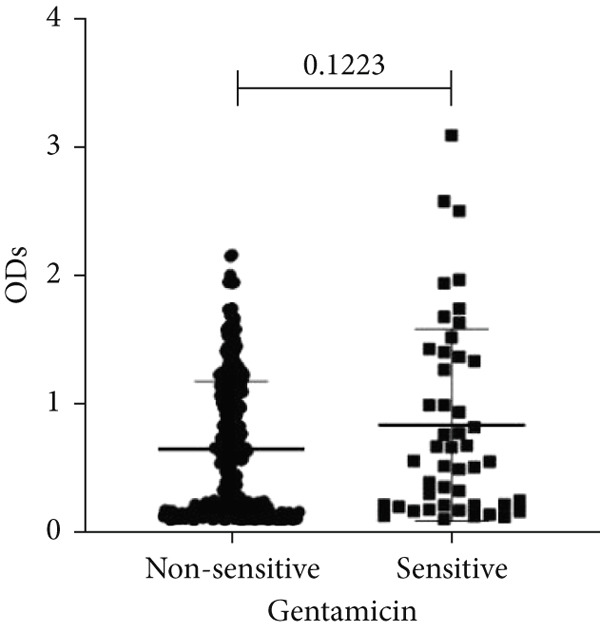
(b)

**Figure:**
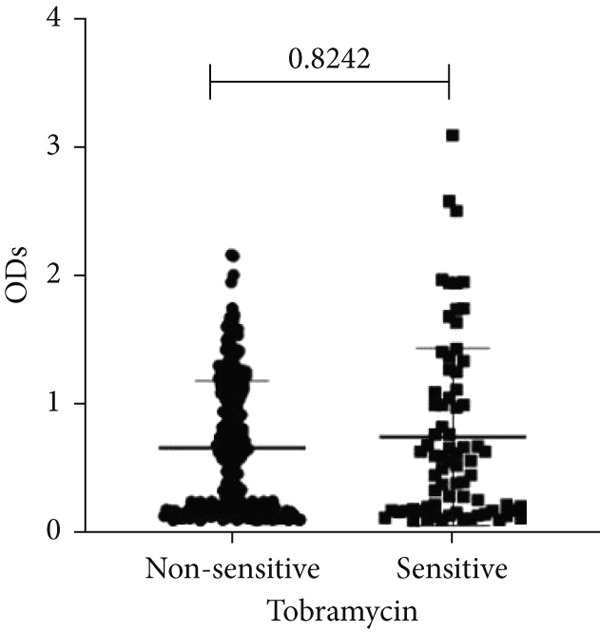
(c)

**Figure:**
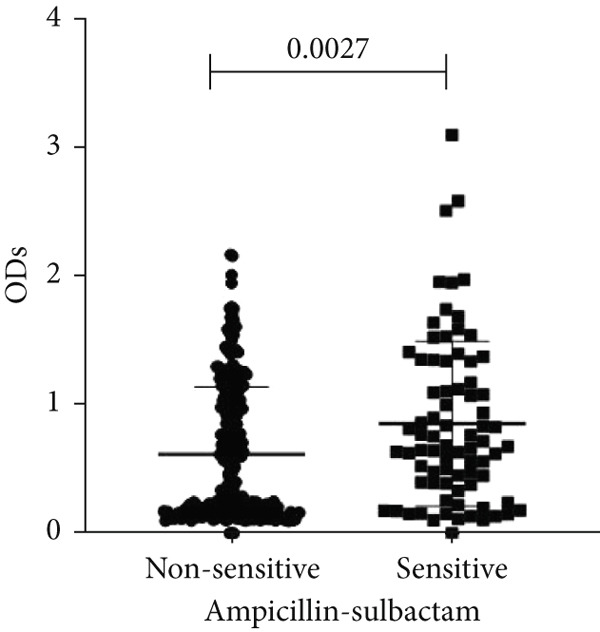
(d)

**Figure:**
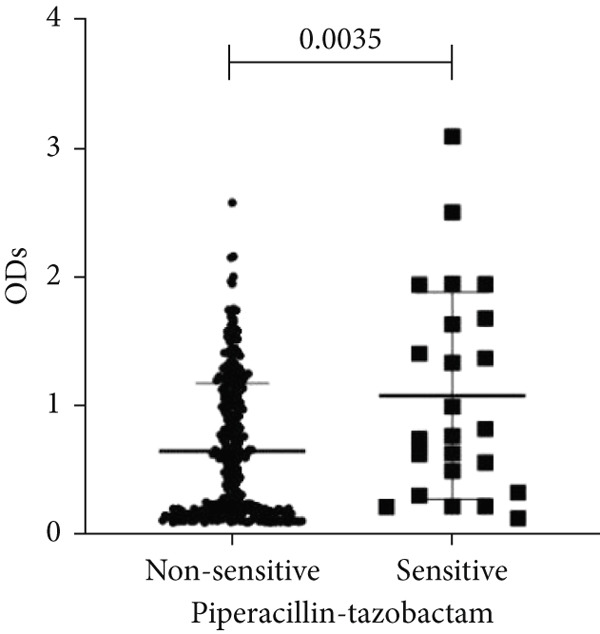
(e)

**Figure:**
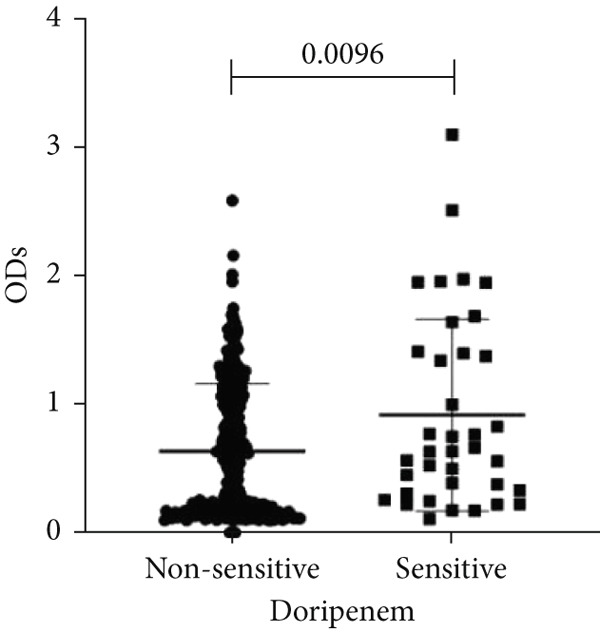
(f)

**Figure:**
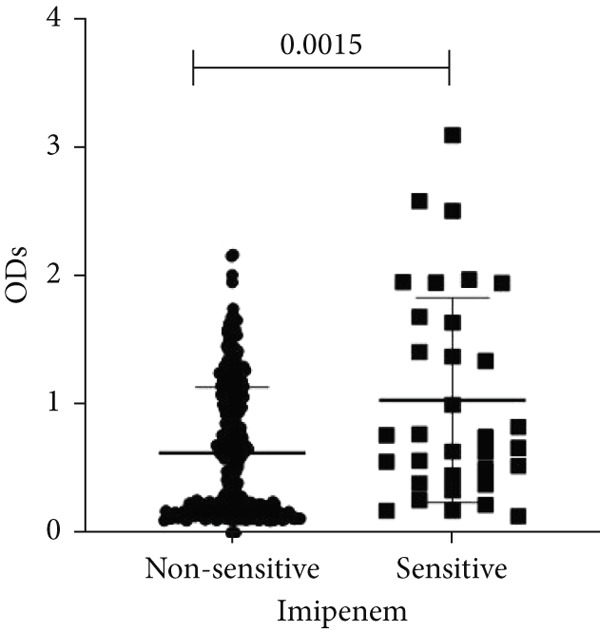
(g)

**Figure:**
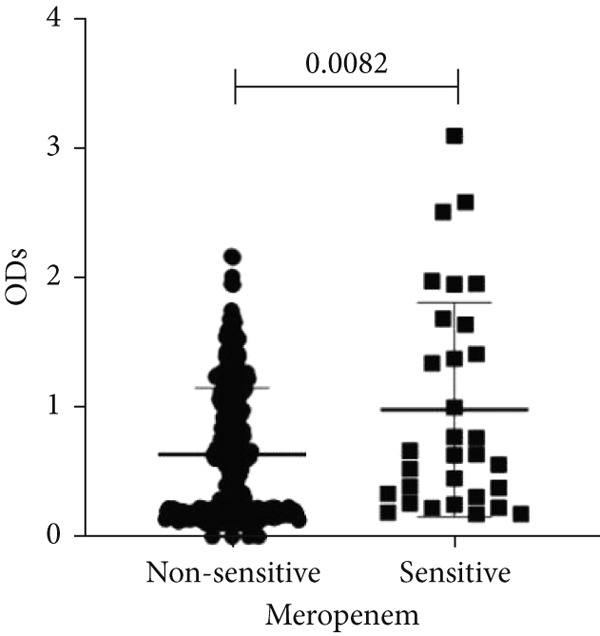
(h)

**Figure:**
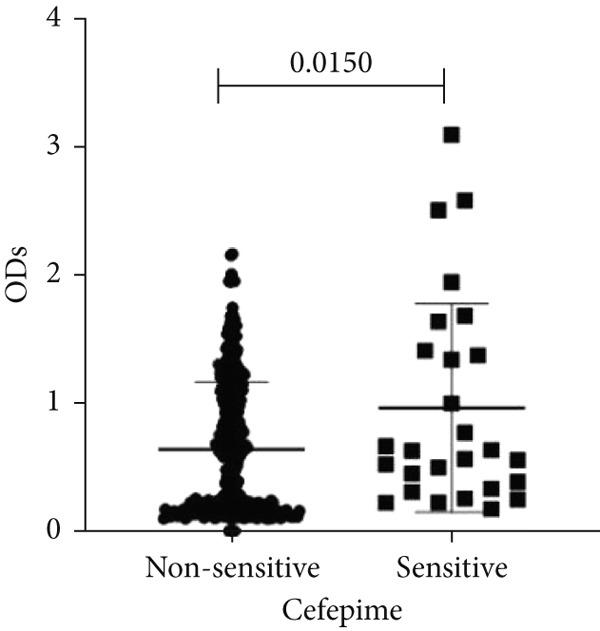
(i)

**Figure:**
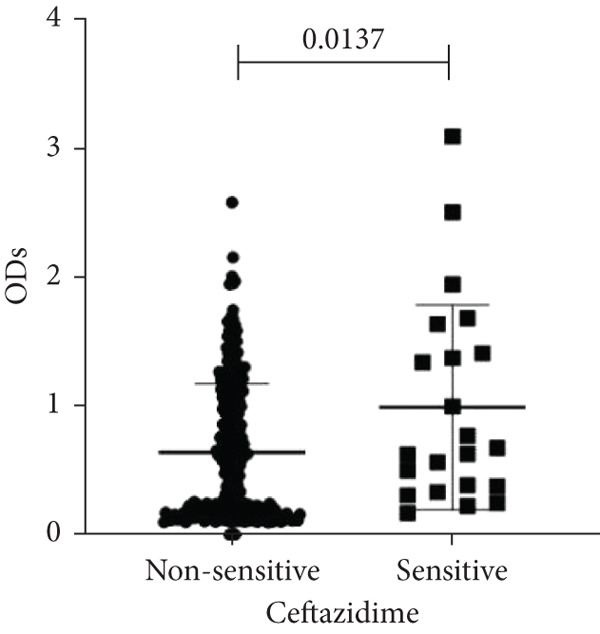
(j)

**Figure:**
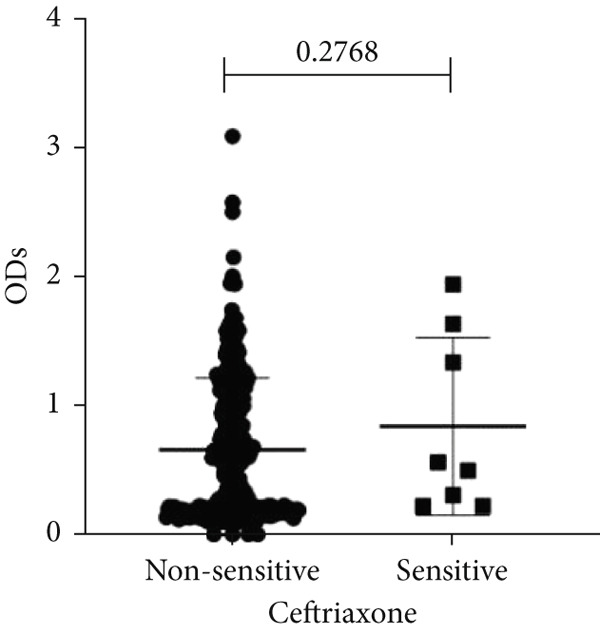
(k)

**Figure:**
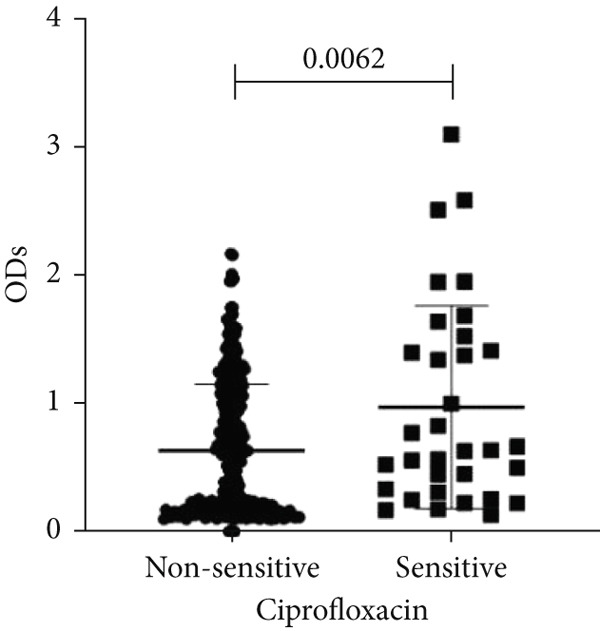
(l)

**Figure:**
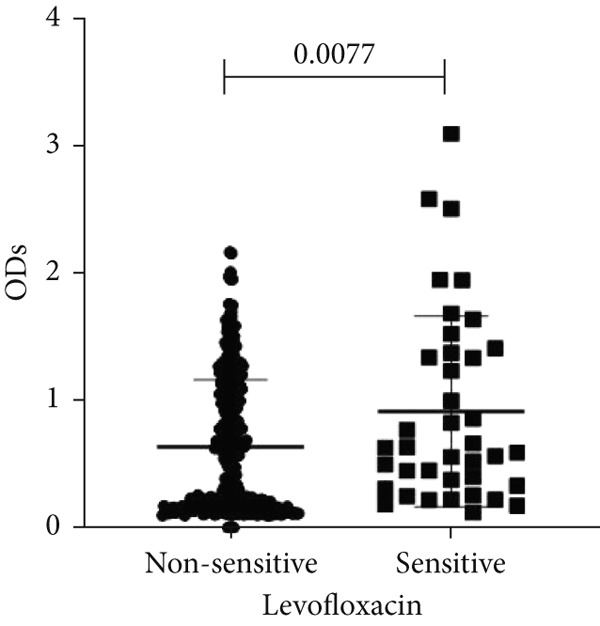
(m)

**Figure:**
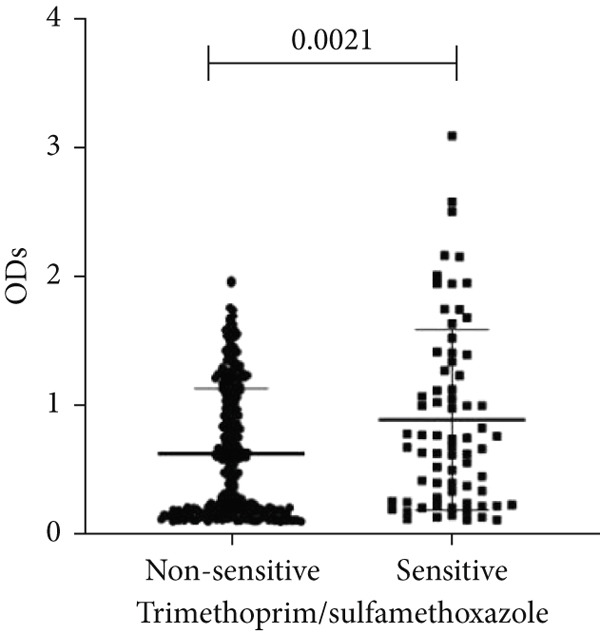
(n)

**Figure:**
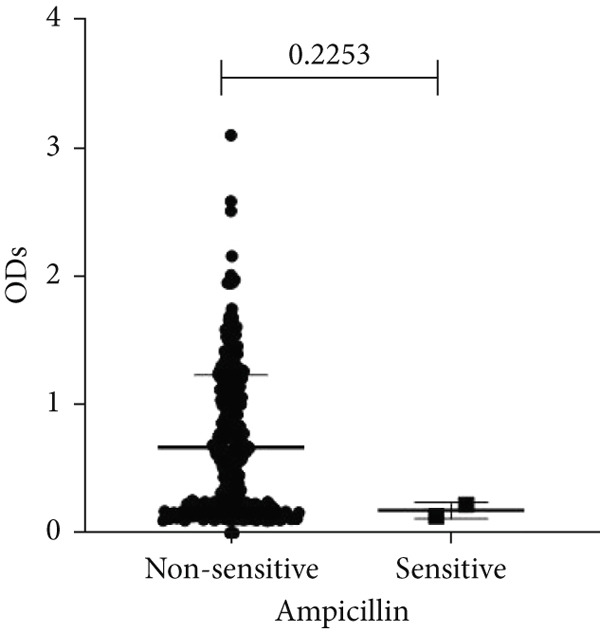
(o)

**Figure:**
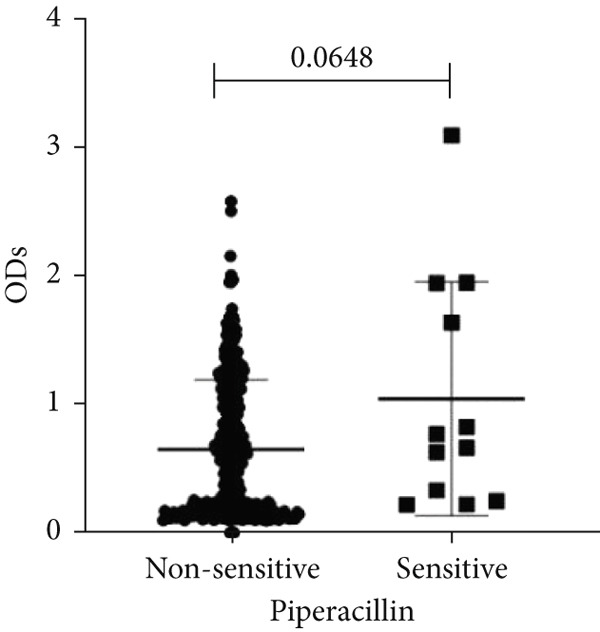
(p)

**Figure:**
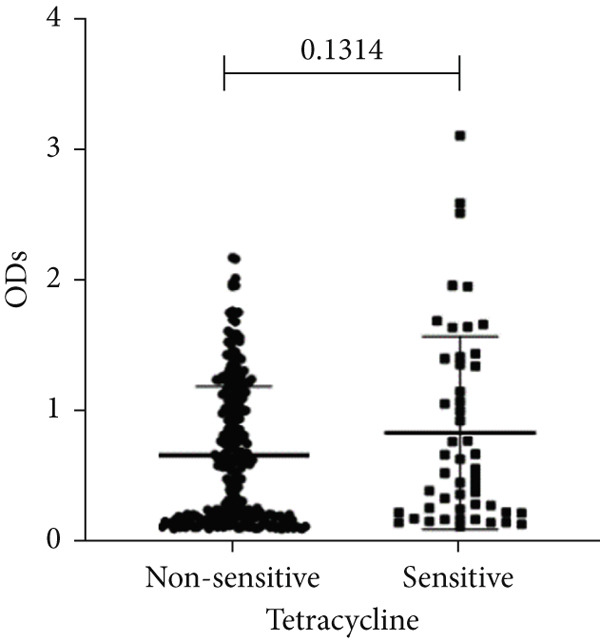
(q)

## 4. Discussion

In our multicenter study, most *A. baumannii* isolates investigated were multidrug resistant, and some exhibited resistance to 16 antibiotics, including carbapenems. Carbapenem resistance was high overall (88.7%) and higher in ICU isolates (94.4%) than in non‐ICU isolates (83.8%), aligning with international trends and reinforcing concerns about stewardship and infection–prevention practices in high‐risk settings [24–29]. We also observed high resistance to *β*‐lactams and fluoroquinolones, which is consistent with prior reports [30–35]. The majority of the isolates (81.2% overall) were classified as XDR, including 86.7% of ICU isolates. The XDR phenotype also dominated across each participating hospital, suggesting endemic nosocomial transmission and cumulative selection from year‐over‐year antibiotic exposure. The high prevalence of XDR isolates among ICU patients underscores risks of empirical therapy failure and increased mortality, and it raises concern that ongoing acquisition of resistance determinants could transform some XDR strains toward PDR if selective pressure remains high.

In addition to genetic resistance, biofilm formation is a key virulence factor that allows *A. baumannii* to persist on medical devices and host tissues, contributing to nosocomial infections. Most isolates were able to form biofilms, with 44.3% producing strong biofilms, consistent with the established role of biofilms in device‐associated infections [15, 36, 37] and their documentation across clinical and environmental *A. baumannii* niches [38–42]. Although strong biofilm formation was more prevalent in ICU isolates (60.7%) than in non‐ICU wards (30.5%), analysis of the biofilm formation data showed no significant shift in the overall distribution between ICU and non‐ICU groups (*p* = 0.145) and no difference by sex (*p* = 0.786). Taken together, these data indicate that ICU settings harbor a higher frequency of strong biofilm producers rather than a uniform elevation in biofilm across all isolates. Clinically, this phenotype distribution emphasizes the need for strict ventilator care and early device replacement. Furthermore, infection prevention measures must be maintained across all wards, as the baseline prevalence of biofilm formation remains high throughout the hospital.

An important finding is the inverse correlation between resistance and the ability to form biofilms. Isolates susceptible to 10 out of the 17 antibiotics tested, including ampicillin–sulbactam, doripenem, imipenem, meropenem, SXT, TZP, levofloxacin, cefepime, ceftazidime, and ciprofloxacin, showed a higher tendency to form biofilms. The same trend was observed within ICU isolates for all antibiotics except ceftriaxone. When comparing antibiotic susceptibility phenotypes, MDR isolates, but not XDR, showed a higher biofilm tendency. This pattern suggests a potential trade‐off; strains lacking extensive resistance mechanisms may rely more on biofilm formation as an alternative strategy for persistence against antimicrobials and host defenses. While our findings agree with several studies reporting this inverse correlation [43–53], other reports describe a positive correlation [37, 54–64]. Such discrepancies can be attributed to differences in the biofilm assays, the methods used to identify *A. baumannii* isolates, as well as the number of isolates analyzed and their clinical source, or the geographical origin of the isolates. A major strength of this study is the inclusion of 327 *A. baumannii* clinical isolates, which, to our knowledge, no other study has analyzed this large number of isolates, especially isolates recovered from ICU patients. Moreover, our isolates were collected from three different hospitals and recovered from six major types of clinical specimens, minimizing any bias from single‐center endemic strains and allowed us to study the contribution of the strains′ site of collection and hospital source on their ability to form biofilm.

To the best of our knowledge, our study is the first to demonstrate a significant association between the clinical source of infection and biofilm capacity. Isolates recovered from normally sterile sites, such as blood and CSF, exhibited greater ability to form biofilms than isolates from sputum, urine, and wounds. In the bloodstream or CSF, where host immunity is the primary threat, robust biofilm may protect bacterial communities from phagocytosis and complement‐mediated killing, supporting biofilm as a virulence determinant in invasive infection. This finding changes our understanding of biofilm from being solely a mechanism for surface persistence to being an offensive mechanism for invasion of deeper tissues. Although some studies have reported stronger biofilms in other specimen types [36, 37, 56, 65, 66], our larger multicenter sampling reduces single‐source bias and provides a rationale for designing prevention and empiric therapy by both hospital and infection site. We also noted variation in biofilm formation among the three hospitals; isolates from KAUH showed lower biofilm capacity and a lower prevalence of XDR than isolates from BH and ZH, suggesting that local factors such as antibiotic prescribing patterns, infection control protocols, and the circulation of specific endemic clones shape the phenotypes of these survival traits.

In conclusion, most *A*. *baumannii* isolates exhibited both high antibiotic resistance and high biofilm capacity, with many classified as XDR and most producing biofilm. Across several antibiotics, isolates with stronger biofilm tendency were more often susceptible, indicating an inverse biofilm–resistance relationship. Results also varied by hospital and by specimen source, especially from blood and CSF. Clinically, pairing routine susceptibility testing with a simple microtiter biofilm assay can help find patients at risk of persistent device‐associated infection, support earlier device intervention, optimize treatment duration, and guide site‐specific prevention and evaluation of the use of anti‐biofilm agents alongside antibiotics for systemic therapy.

## Disclosure

All authors have reviewed and approved the final version of the manuscript.

## Conflicts of Interest

The authors declare no conflicts of interest.

## Author Contributions

All authors have contributed significantly to the writing and preparation of the manuscript.

## Funding

This work was supported by the Deanship of Research, Jordan University of Science and Technology (10.13039/501100019004) (20180475).

## Data Availability

The data that support the findings of this study are available from the corresponding author upon reasonable request.
